# Incidence, risk factors, and adverse outcomes of acute kidney injury in very premature neonates: a single center experience

**DOI:** 10.3906/sag-2012-348

**Published:** 2021-10-21

**Authors:** Nuran ÜSTÜN

**Affiliations:** 1 Department of Pediatrics, Faculty of Medicine, İstanbul Medeniyet University, İstanbul Turkey

**Keywords:** Acute kidney injury, very preterm infants, mortality, neonatal KDIGO

## Abstract

**Background/aim:**

Acute kidney injury (AKI) is a serious morbidity in premature neonates. The aim of this study was to determine the incidence of AKI and to evaluate its impact on morbidity and mortality in very premature infants.

**Materials and methods:**

This retrospective cohort study was conducted in the neonatal intensive care unit (NICU). A total of 410 preterm infants who were born before 32 gestational weeks were screened and 318 were included in this analysis. AKI was defined according to the modified neonatal Kidney Disease: Improving Global Outcomes criteria.

**Results:**

The incidence of AKI was 32.1% (102/318). Regression analyses revealed that lower gestational age, vasopressor use, and hemodynamically significant patent ductus arteriosus were significantly associated with an increased risk for AKI. After adjustment for potential confounders, those with AKI had a higher risk of death before 36 weeks of corrected gestational age (adjusted hazard ratio: 3.02, 95% confidence interval 1.47– 6.22). Additionally, the AKI group had a higher rate of bronchopulmonary dysplasia (BPD) (46% vs. 24%, p < 0.001) and longer hospital stay with a mean difference of 38 days.

**Conclusion:**

AKI is common in very premature neonates and associated with higher mortality, longer hospital stay, and BPD. Identification of risk factors and preventive strategies for AKI may improve the outcomes in this vulnerable population.

## 1. Introduction

Nephrogenesis is completed at about 36 weeks of gestation. Premature neonates are prone to develop acute kidney injury (AKI) due to kidney immaturity and high risk of exposure to hemodynamic changes, infections and nephrotoxic medications [1,2]. Surviving premature infants are also at risk for chronic kidney disease later in life [2]. Studies on pediatric and adult patients have shown that even a small change in serum creatinine (SCr) was associated with increased mortality [3,4]. Similar associations were noted for premature infants in recent studies [5–10].

The incidence of AKI in preterm neonates has been reported to range from 12 to 48%. This large variation could be the result of quite different definitions of AKI [2]. Newer AKI definitions such as modified pediatric risk, injury, failure, loss and end-stage renal disease (pRIFLE) [11], acute kidney injury network (AKIN) [12] and Kidney Disease: Improving Global Outcomes (KDIGO) [13] have made progress in diagnosing AKI in the pediatric and adult population. These definitions are based on percentage of increase in serum creatinine (SCr) and oliguria. To adopt a standardized AKI definition for neonates, a modified KDIGO definition was proposed by Jetton and Askenazi (Table 1) [14]. However, there are limited studies using this new definition of AKI in premature infants.

**Table 1:** Modified neonatal acute kidney injury Kidney Disease: Improving Global. Outcomes criteria

Stage	Serum creatinine (SCr)	Urine output
0	No change in SCr or increase < 0.3 mg/dL	≥ 0.5 mL/kg/h
1	SCr increase ≥ 0.3 mg/dL within 48 h orSCr increase ≥ 1.5 to 1.9 referencea SCr within 7 d	< 0.5 mL/kg/h for 6-12 h
2	SCr increase ≥ 2 to 2.9 referencea SCr	< 0.5 mL/kg/h for ≥ 12 h
3	SCr increase ≥ 3 referencea SCr or SCr ≥ 2.5 mg/dL or receipt of dialysis	< 0.3 mL/kg/h for ≥ 24 h or anuria for ≥ 12 h

a : Reference SCr was defined the lowest previous SCr.

The aim of this study was to investigate the incidence and risk factors of AKI in very premature newborns using the neonatal KDIGO definition, and to evaluate the relationship between AKI and adverse neonatal outcomes.

## 2. Materials and methods

### 2.1. Study population

**Figure 1:**
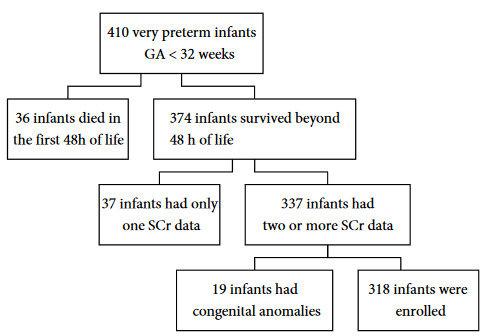
Flowchart of the selection of patients. GA, gestational age; SCr, serum creatinine.

This retrospective cohort study was performed in a level 3 neonatal intensive care unit (NICU) from January 2017 to December 2019. A total of 410 infants who were born before 32 gestational weeks were screened. Thirty-six infants who died within the first 48 h of life, 19 infants with major congenital anomalies, and 37 infants with insufficient SCr data were excluded from the study. Consequently, 318 infants were available for this analysis (Figure 1). This study was approved by the Hospital Ethics Committee, on 1 June 2020, with a registration number of 2020/0413.

### 2.2. Renal function tests

For all study patients, SCr values measured as a part of routine care were recorded. In our NICU, a routine biochemistry panel including SCr was obtained at least every 2 days during the first week of life in these infants; then once a week. In case of clinical deterioration, SCr levels were monitored for at least the next 48 h. AKI was diagnosed according to the modified neonatal KDIGO criteria which takes into account the increase in SCr compared to a reference value rather than a single cutoff value (Table 1) [14]. Based on these criteria, AKI was defined as a SCr rise of ≥ 0.3 mg/dL within 48 h or ≥1.5 times the reference value within 7 days. Creatinine values obtained during the first two days of life may reflect maternal creatinine levels [15] and the postnatal transition period [16]. Therefore, reference SCr was defined as the lowest previous SCr value after the first 48 h of age. Urine output criteria were not included because it was difficult to measure in preterm infants. In cases with more than one AKI episode, the highest AKI stage was included in the analysis.

### 2.3. Data collection

Hospital electronic medical records and patient files were reviewed for data collection. Maternal characteristics included diagnosis of preeclampsia, diabetes, prolonged rupture of membranes (PROM) for more than 18 h, chorioamnionitis, and the administration of antenatal steroids. Chorioamnionitis was defined as the presence of fever and treatment with intravenous antibiotics. Demographic factors included gestational age (GA), birth weight, sex, delivery mode, Apgar score at 5 min, and small for gestational age status. The following clinical characteristics were also documented: need for vasopressors and high frequency ventilation, hemodynamically significant patent ductus arteriosus (HSPDA), any grade intraventricular hemorrhage (IVH), culture positive sepsis, and use of nephrotoxic medication (vancomycin and gentamicin).

In our unit, echocardiography was performed by a pediatric cardiologist in very premature newborns between the 3rd to 5th days of life. As per our unit policy, ibuprofen was used as the first choice for patients with HSPDA, and paracetamol treatment was given if there is a contraindication to the use of cyclooxygenase inhibitors. Surgical ligation was performed in patients with HSPDA despite three cycles of medical therapy. All patients were evaluated in the 3 days of life for IVH and then weekly by a neonatologist.

The primary outcome was survival to discharge or 36 weeks of corrected gestational age (CGA). Bronchopulmonary dysplasia (BPD) and length of hospital stay were evaluated as secondary outcomes. BPD was defined as oxygen dependency at 36 weeks of CGA.

### 2.4. Statistical analysis

SPSS 23.0 for Windows program was used for data analysis. The distribution of the sample was checked by the Shapiro–Wilk test. Normally distributed data were represented by the mean and standard deviation (SD), whereas skewed data was represented by the median and interquartile range (IQR). Continuous data were compared using the Student’s t-test or Mann–Whitney U test. Chi-squared tests were used for the evaluation of categorical data. Variables with p < 0.2 on univariate analysis were included in a multivariate logistic regression model. A stepwise backward selection approach was used to identify independent risk factors for AKI. Cox regression analysis was performed for mortality before 36 weeks of CGA. Variables with p < 0.2 in the univariate test were included in the multivariate model and the stepwise backward selection was performed to determine independent predictors of mortality. Collinearity among the covariates was tested.

## 3. Results

The study included 164 males (51.6%) and 154 females (48.4%). The mean gestational age and birth weight were 28.1 ± 2.3 weeks (23–31 weeks) and 1129.6 ± 320.6 g (410–1910 g), respectively. AKI was diagnosed in 102 of 318 patients, the incidence of AKI was 32.1%. When classified according to the modified KDIGO criteria, 68 infants (67%) were stage 1, 19 infants (19%) were stage 2 and 15 infants (15%) were stage 3. The majority of infants with AKI had GA < 28 weeks and birth weight < 1000 g (Tables 2 and 3). AKI episodes mostly occurred during the first week of life (73 of 102) (median: 5 days, IQR: 5–7 days).

**Table 2:** Incidence of AKI stratified by gestational age.

AKI	< 750 g (n = 63)	750–1000 g(n = 70)	11000–1250 (n = 97)	> 1250 g(n = 88)	Total(n = 318)	p value
No	23 (36.5%)	35 (50%)	78 (80.4%)	80 (90.1%)	216 (67.9%)	< 0.001
Stage 1	25 (39.7%)	21 (30%)	16 (16.5%)	6 (6.8%)	68 (21.4%)	
Stage 2	8 (12.7%)	8 (11.4%)	3 (3.1%)	0 (0%)	19 (6%)	
Stage 3	7 (11.1%)	6 (8.5%)	0 (0%)	2 (2.3%)	15 (4.7%)	

**Table 3:** Incidence of AKI stratified by birth weight.

AKI	< 26 weeks(n = 76)	26–27 weeks(n = 76)	28–29 weeks (n = 87)	30–31 weeks (n = 79)	Total(n = 318)	p value
No	39 (51.3%)	42 (55.3%)	69 (79.3%)	66 (83.5%)	216 (67.9%)	< 0.001
Stage 1	23 (30.3%)	22 (28.9%)	12 (13.8%)	11 (13.9%)	68 (21.4%)	
Stage 2	8 (10.5%)	6 (7.9%)	4 (4.6%)	1 (1.3%)	19 (6%)	
Stage 3	6 (7.9%)	6 (7.9%)	2 (2.3%)	1 (1.3%)	15 (4.7%)	

Patient characteristics of the infants with and without AKI are compared in Table 4. Maternal factors including diabetes, preeclampsia, PROM, chorioamnionitis and antenatal steroid use were not significantly different between infants with and without AKI. Infants with AKI had lower GA, birth weight and 5 min Apgar scores. In univariate analysis, vasopressor use, HSPDA, IVH, need for high frequency ventilation, culture proven sepsis, and exposure to nephrotoxic antibiotics were all significantly associated with AKI. After controlling for all confounders with univariate p < 0.2; lower GA, vasopressor use, and HSPDA were identified as risk factors for AKI (Table 5).

**Table 4:** Demographic and clinical characteristics of patients with and without AKI.

	No AKI(n = 216)	AKI(n = 102)	p value
Maternal diabetes	20 (9.3%)	11 (10.8%)	0.669
Preclampsia	63 (29.2%)	22 (21.6%)	0.153
PROM	40 (18.5%)	24 (23.5%)	0.298
Chorioamnionitis	18 (8.3%)	12 (11.8%)	0.329
Antenatal steroid	161 (74.5%)	70 (68.6%)	0.270
Gestational age (week)*	29.1 ± 2.1	27.5 ± 2.2	< 0.001
Birth weight, (g)*	1320.0 ± 253.4	894.5 ± 251.9	< 0.001
Cesarean delivery	158 (73.1%)	66 (64.7%)	0.124
Male sex	115 (53.2%)	49 (48%)	0.386
SGA	25 (11.6%)	16 (15.7%)	0.307
Apgar at 5 min**	8 (6, 9)	7 (5, 8)	< 0.001
Vasopressor use	22 (10.2%)	37 (36.3%)	0.001
Culture proven sepsis	32 (14.8%)	25 (24.5%)	0.035
HSPDA	34 (15.7%)	41 (40.2%)	< 0.001
HFV	45 (20.8%)	34 (32.4%)	0.016
IVH	27 (12.5%)	23 (22.5%)	0.022
Nephrotoxic medication	171 (79.2%)	92 (90.2%)	0.015

* : mean ± standard deviation.

**Table 5:** Results of logistic regression of risk factors for AKI.

Variable	Odds ratio ( 95% CI)*	p value
Gestational age	0.68 (0.59–0.78)	< 0.001
HSPDA	1.92 (1.04–3.55)	0.037
Vasopressor use	2.68 (1.37–5.25)	0.004

*: Adjusted for gestational age, cesarean delivery, Apgar scores at 5 min, need for high frequency ventilation, vasopressor use, intraventricular hemorrhage, hemodynamically significant patent ductus arteriosus, culture proven sepsis, nephrotoxic medication use and maternal preeclampsia.

**Figure 2:**
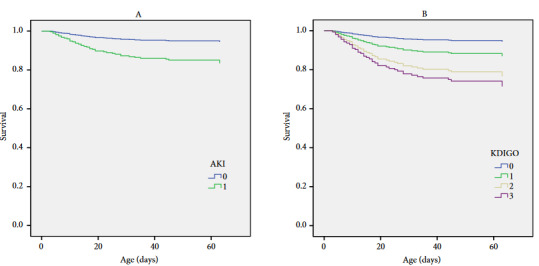
Survival curve for patient with AKI.

Out of the 318 very premature infants, 41 (12.9) died before 36 weeks of CGA. Infants with AKI had a higher rate of mortality (29% vs. 5%). In univariate analysis, AKI was strongly associated with mortality with a crude hazard ratio (HR) of 5.73 (95% CI 2.78–11.78; p < 0.001). Other variables significantly associated with mortality included GA, birth weight, vasopressor use, high frequency ventilation, HSPDA, IVH, and culture proven sepsis (Table 6). After controlling for confounding variables with univariate p < 0.2; AKI remained a predictor of mortality (adjusted HR: 3.01, 95% CI: 1.47–6.22, p = 0.003) (Table 6, Figure 2A). When stratified by stage, all AKI stages including stage 1 were associated with mortality (Table 6, Figure 2B). Among the 277 survivors, patients with AKI had a higher rate of BPD (46% vs. 24%, p < 0.001) and longer hospital stay (median: 84 days, IQR: 67.5–103.5 days vs. median: 45 days, IQR: 33–60 days, p < 0.001). After adjustment for GA and birth weight, AKI was associated with a higher rate of BPD (adjusted OR 1.91 95% CI 1.05–3.47, p = 0.033).

**Table 6:** Predictors of mortality before 36 weeks of corrected gestational age.

	Crude hazard ratio(95% CI)	p value
Univariate model		
Gestational age (week)	0.61 (0.52–0.72)	< 0.001
Birth weight (100 g)	0.73 (0.65–0.83)	< 0.001
Male sex	1.44 (0.78–2.67)	0.241
Apgar at 5 min,	0.82 (0.66–1.01)	0.065
Small for gestational age	1.71 (0.79–3.71)	0.174
Vasopressor use	8.35 (4.17–16.71)	< 0.001
Culture proven sepsis	4.78 (2.47–9.25)	< 0.001
Significant patent ductus arteriosus	2.83 (1.53–5.25)	0.001
High frequency ventilation	2.37 (1.27–4.41)	0.007
Intraventricular hemorrhage	3.00 (1.62–5.55)	< 0.001
Nephrotoxic medication	2.11 (0.51–8.78)	0.305
Any acute kidney injury	5.73 (2.78–11.78)	< 0.001
Acute kidney injury stage 1	4.36 (1.96–9.69)	< 0.001
Acute kidney injury stage 2	9.68 (3.91–23.96)	< 0.001
Acute kidney injury stage 3	7.29 (2.63–20.21)	< 0.001
Maternal diabetes	1.00 (0.44–2.29)	0.982
Preclampsia	0.53 (0.20–1.43)	0.213
Prolonged rupture of membrane	1.86 (0.96–3.60)	0.062
Chorioamnionitis	1.64 (0.69–3.89)	0.265
Cesarean delivery	0.79 (0.42–1.50)	0.483
Multivariate model#		
Any acute kidney injury	3.02 (1.47–6.22)	0.003
Acute kidney injury stage 1	2.37 (1.08–5.20)	0.032
Acute kidney injury stage 2	4.27 (1.48–12.31)	0.007
Acute kidney injury stage 3	5.63 (2.07–15.30)	0.001

Cox proportional hazard ratios for GA per week increase, birth weight per 100 g increase, Apgar scores per point increase, and categorical variables compared with any AKI, stage 1 AKI, stage 2 AKI, stage 3 AKI, and absence of AKI.# : Control for 5 min Apgar score, small for gestational age, vasopressor use. culture proven sepsis, significant PDA, need for high frequency ventilation, IVH, and PROM

## 4. Discussion

In this study, we demonstrated that one-third of very premature infants developed AKI. Perinatal factors including lower GA, vasopressor use, and presence of HSPDA were identified as significant risk factors for AKI. AKI was strongly associated with mortality before 36 weeks of CGA. 

The incidence of AKI in very premature neonates in our NICU was 32.1%. However, not including urine output criteria in AKI diagnoses might result in an underestimation of this incidence. Using the same KDIGO SCr criteria, Stojanovic et al. [9] found that AKI developed at a rate of 26% in all premature infants and 39% in very low birth weight (VLBW, < 1500 g) premature infants. In the multicenter, multinational AWAKEN cohort study [17], the incidence of AKI was 48% in newborns with GA < 28 weeks and 18% in newborns with GA > 28 weeks. In our study, AKI occurred in 71 (50%) of 142 infants with GA < 28 weeks. However, in another study by Stojanovic et al. [8], they showed that 44% (85/195) of premature infants had AKI. Abdelaal et al. [18] performed a study on 60 premature infants with respiratory distress and found that AKI developed at a rate of 40%. Although similar diagnostic criteria were used in our study and the above studies, there was a wide difference in the incidence of AKI. This can be attributed to the differences in the patient characteristics in terms of risk factors, overall sickness, and management methods. Similar to previous studies [17], we found that majority (67%) of AKI cases were stage 1.

We found that a lower GA, vasopressor use, and the presence of HSPDA were independent risk factors for AKI in very preterm neonates. The incidence of AKI was inversely proportional to the gestational age. This might be explained by the increasing risk of exposure to AKI risk factors. Whereas, after controlling all possible risk factors, a lower GA remained as an independent risk for AKI. 

PDA is serious morbidity associated with an increased risk of IVH, BPD, and death in preterm infants [19]. An HSPDA with shunting of blood across the ductus results in compromised systemic perfusion and pulmonary overflow. Systemic hypoperfusion could lead to renal injury. Ibuprofen, a nonsteroidal antiinflammatory (NSAID) medication, used to treat PDA could worsen the problem [20]. On the other hand, NSAID exposure might have beneficial renal effects as a result of ductal closure [21]. Our data suggested that HSPDA was significantly associated with AKI. However, we did not analyze the effect of ibuprofen on the development of AKI separately. Future studies on the relationships between AKI and NSAID exposure in PDA treatment would be useful.

The need for vasopressor support reflects the higher disease severity in newborns [22]. Our results supported that the need for vasopressor support was significantly associated with AKI. Although vasopressor use and HSPDA were associated with AKI, their causative effect needs to be explained. As a marker of kidney function, a rise in SCr level occurs late after kidney injury. Also, HSPDA or severe hypotension requiring vasopressors usually develops during the first week of life as AKI episodes. Thus, kidney injury might promote these comorbidities. 

Previous studies have shown relationship between neonatal AKI and vancomycin use or sepsis [8,23]. Nephrotoxicity has been reported in 2 to 20% of newborns treated with vancomycin [8]. However, a cohort study using propensity score matching reported no significant association between vancomycin and AKI [24]. In our study, culture positive sepsis and nephrotoxic antibiotics use were more frequent in infants with AKI, but after adjustment other covariates, no significant association was observed between AKI and sepsis or nephrotoxic antibiotic use. However, we did not have data on the duration of nephrotoxic antibiotic exposure which might affect our results. These variations can also be explained by the differences in patient characteristics in studied populations.

Prenatal factors such as maternal diabetes and preeclampsia have been identified as protective factors for neonatal AKI [17,22]. In our study, although not significant, the incidence of AKI was lower in infants of mothers with preeclampsia. Other prenatal factors did not differ between infants with and without AKI. Further case-control studies with a larger sample size are required to explain the contribution of these factors in AKI development.

The impact of AKI in premature infants on survival and other outcomes has become the focus of interest in recent studies. In a case-control study, Askenazi et al. [5] showed a two-fold increase in the probability of death for a small increase (1 mg/dL) in SCr. In a study of 1461 premature infants (GA of < 33 weeks) conducted by Bruel et al. [25], AKI was associated with higher mortality. In our study, AKI reduced overall survival at 36 weeks of CGA in very premature infants (adjusted HR 3.0). Particularly, 26% of infants with stage 1 AKI died before 36 weeks of CGA and stage 1 AKI was associated with increased mortality. Lassnigg et al. [26] previously demonstrated that minimal elevation of SCr levels after cardiac surgery was associated with serious implications. These findings suggest that even slightly elevated SCr in seriously ill patients require careful evaluation and long-term follow-up. As an important indicator of morbidity, the duration of hospital stay was longer in infants with AKI. Additionally, these patients had higher rates of BPD. The association between AKI and prolonged ventilation has been described in critically ill pediatric patients [27]. Starr et al. [28] retrospectively evaluated 546 very premature infants and reported a relationship between AKI and the development of BPD. Animal-based researches also have shown that the kidney has an important role in the stability of systemic inflammation and cytokine balance and that kidney injury may result in an inflammatory reaction in the lung [29,30]. Recent studies reported that AKI can lead to chronic kidney disease and an impaired immune system later in life [31]. Thus, serial follow-up of kidney functions of surviving premature neonates is necessary. 

Our study has some limitations. First, since SCr level was not measured daily, the incidence and severity of AKI might be underestimated. Second, because of the retrospective design of the study, confounders related to AKI and outcomes may not be effectively eliminated. Third, SCr is not a good marker of renal injury and estimated glomerular filtration rate since SCr generally does not change until a 25%–50% decrease in kidney function [32–34]. The novel biomarkers that have been proposed to estimate AKI [35] were not used in our study. Further prospective studies with novel biomarkers of renal injury in addition to urine output are needed to improve the detection of renal injury. 

In conclusion, the results of our study show that AKI is a common condition in very premature neonates and can reduce survival at 36 weeks of CGA. Lower gestational age, the presence of HSPDA, and vasopressor use were found as independent risk factors for AKI. Surviving infants after AKI had a higher rate of BPD and longer hospital stay. Therefore, identification of risk factors and preventive strategies for AKI may ameliorate the outcomes in this vulnerable population. The effect of AKI on long-term kidney function and overall survival remains to be elucidated.

## Informed consent

The Ethics Committee of Göztepe Training and Research Hospital approved the study protocol on 1 June 2020, with a registration number of 2020/0413 and waived the need for informed consent or parental permission.
